# Tissue signatures influence the activation of intrahepatic CD8^+^ T cells against malaria sporozoites

**DOI:** 10.3389/fmicb.2014.00440

**Published:** 2014-08-22

**Authors:** Alexandre Morrot, Maurício M. Rodrigues

**Affiliations:** ^1^Departamento de Imunologia, Instituro de Microbiologia, Universidade Federal do Rio de JaneiroRio de Janeiro, Brazil; ^2^Departmento de Microbiologia, Imunologia e Parasitologia, Universidade Federal de São Paulo-Escola Paulista de MedicinaSão Paulo, Brazil

**Keywords:** malaria liver stages, CD8^+^ T cells, vaccine, migration and invasion, hepatocytes

## Abstract

*Plasmodium* sporozoites and liver stages express antigens that are targeted to the MHC-Class I antigen-processing pathway. After the introduction of *Plasmodium* sporozoites by *Anopheles* mosquitoes, bone marrow-derived dendritic cells in skin-draining lymph nodes are the first cells to cross-present parasite antigens and elicit specific CD8^+^ T cells. One of these antigens is the immunodominant circumsporozoite protein (CSP). The CD8^+^ T cell-mediated protective immune response against CSP is dependent on the interleukin loop involving IL-4 receptor expression on CD8^+^ cells and IL-4 secretion by CD4^+^ T cell helpers. In a few days, these CD8^+^ T cells re-circulate to secondary lymphoid organs and the liver. In the liver, the hepatic sinusoids are enriched with cells, such as dendritic, sinusoidal endothelial and Kupffer cells, that are able to cross-present MHC class I antigens to intrahepatic CD8^+^ T cells. Specific CD8^+^ T cells actively find infected hepatocytes and target intra-cellular parasites through mechanisms that are both interferon-γ-dependent and -independent. Immunity is mediated by CD8^+^ T effector or effector-memory cells and, when present in high numbers, these cells can provide sterilizing immunity. Human vaccination trials with recombinant formulations or attenuated sporozoites have yet to achieve the high numbers of specific effector T cells that are required for sterilizing immunity. In spite of the limited number of specific CD8^+^ T cells, attenuated sporozoites provided multiple times by the endovenous route provided a high degree of protective immunity. These observations highlight that CD8^+^ T cells may be useful for improving antibody-mediated protective immunity to pre-erythrocytic stages of malaria parasites.

## Introduction

Malaria is a vector-born, parasitic illness caused by infection with *Plasmodium* spp. More than 3 billion individuals live in the endemic areas, which are mainly in tropical areas of the world. The disease causes approximately 600,000 annual deaths and most individuals are children living in Africa. In adults, pregnant women are vulnerable to malaria as pregnancy reduces their immunity. This problem has long been neglected but the maternal malaria causes an increased risk of spontaneous abortion, premature delivery and low birth weight of the fetus (Cotter et al., 2013; WHO Malaria Policy Advisory Committee and Secretariat, 2013; White et al., 2014). *Plasmodium* parasites have a complex life cycle, and the disease begins by the bite of an infected female Anopheline mosquito carrying the sporozoite form, which is the pre-erythrocytic stage of *Plasmodium* parasites. *Plasmodium* sporozoites are released from the secretory duct of the vector salivary gland where they develop and are injected into the bite site of the skin during blood meals. In the epidermis, the sporozoites actively cross the capillary vessels to enter the bloodstream from where they reach the liver (Khan and Waters, 2004; Miller et al., 2013).

After reaching the liver parenchyma, the sporozoites invade hepatocytes to divide and produce thousands of merozoites, which are the erythrocytic forms of *Plasmodium* parasites (Prudêncio et al., 2006). Parasite growth inside hepatocytes causes host cellular rupture, releasing the merozoites into the bloodstream, where they subsequently invade erythrocytes to initiate a cycle of intra-erythrocytic stage development. In this stage, the *Plasmodium* parasites continuously grow inside red blood cells, causing rupture and re-invasion of healthy erythrocytes and resulting in increased numbers of parasites in the bloodstream every 48 h (Prudêncio et al., 2006; Wright and Rayner, 2014).

Unlike the erythrocytic-stage of *Plasmodium* infection, which is responsible for the clinical symptoms and pathology of the disease, the liver stage of malaria is clinically silent but significantly relevant in the point of view of the host immune defense mechanisms (Frevert and Nardin, 2008). The hepatocyte cells are an obligatory destination for schizogony during the intrahepatic stage, which lasts for 2–7 days, depending on the mammalian host (5–10 days in humans), thus allowing the protective, cell-mediated immune responses to target the reservoirs of parasites in the liver. These hepatic reservoirs are a crucial target for immunological intervention achievements, as efficacious and effective pre-erythrocytic stage immunity would prevent the release of parasites from the hepatocyte and, consequently, the development of clinical disease and transmission of malaria (Doolan and Martinez-Alier, 2006; Duffy et al., 2012). The most clinically advanced malaria vaccine candidates for preventing disease are based on liver-stage *Plasmodium* antigens able to initiate protective immune responses and are expected to target endemic areas of greatest disease burden (Duffy et al., 2012; Birkett et al., 2013).

## *Anopheles* vector transmission of *Plasmodium* parasites in the skin sets the first stage for induction of CD8^+^ T cell-mediated immunity against malaria sporozoites

The sporozoite parasites are differentiated in the salivary glands of the mosquito vector and are inoculated in the vertebrate skin as the mosquito probes to locate the capillary vessels during the blood meal. During this phase, the sporozoites are injected into the skin tissue, a process that ends when the proboscis of the vector reaches the blood circulation and salivation no longer takes place (Vanderberg, 2014). Once in the skin, the sporozoites face a dangerous journey to the liver. They must find their way to hepatocytes, where they develop to the erythrocytic stage. Once the sporozoites enter the skin, the parasites are immediately targeted by innate host immune responses. Besides its own antigenic properties, the saliva proteins of the vector can influence several physiological responses in the skin, as the salivary contents have immunomodulatory properties (Demeure et al., 2005; Beghdadi et al., 2008; Hayashi et al., 2012; Mecheri, 2012).

The tissue trauma generated from the arthropod bite settles the cutaneous immune response behavior at the beginning of infection, as this injury generates endogenous danger signals (danger-associated molecular patterns) that, together with the exogenous PAMPs associated with *Plasmodium* parasites, play a crucial role in the initiation of the immune response (Naik et al., 2000; Gowda, 2007; Erdman et al., 2008; Greene et al., 2009; Fu et al., 2012; Gbédandé et al., 2013). While blood stage parasite PAMPs such as GPI or hemozoin/DNA have been identified (Gowda, 2007; Erdman et al., 2008), sporozoite PAMPs that could play a role in the skin remain to be defined. Thus, parasite-host factors during vector-mediated malaria transmission could be a determinant for activating the innate immune responses that would have an impact in the onset of parasite-specific adaptive immunity (Coban et al., 2007; Franklin et al., 2009; Gowda et al., 2012).

The arthropod vector also plays a role on this battlefield scenario because the *Anopheles* mosquito harbors an associated, symbiotic microbiome that is beneficial to host life related-traits, such as development, fecundity dietary, adaptation to the environment and immunity against pathogenic microorganisms, including *Plasmodium* parasites, in its digestive tract (Dinparast Djadid et al., 2011; Wang et al., 2011; Alvarez et al., 2012; Jiang et al., 2012; Ngwa et al., 2013). Glycosylphosphatidylinositol of *Plasmodium* parasites works as an exogenous PAMP to promote host cellular pro-inflammatory responses, mainly via TLR2 (Gowda, 2007). It is possible that, during the mosquito bite, the sporozoites are injected with an assortment of symbiotic microorganisms into the skin. Therefore, the mosquito microbiome itself could potentiate the innate signaling mechanisms involved in the skin immunity.

Recent studies using an experimental mouse model for malaria infection have substantially increased our comprehension of the host cellular responses that occur in cutaneous tissues during an *Anopheles* mosquito bite. An initial report has shown evidence that mosquito bites promote mast cell degranulation, leading to increased vascular permeability with plasma leakage and leukocyte infiltration into the injured tissue (Depinay et al., 2006). These host responses are initially followed by an accumulation of CD3^+^ and B220^+^ lymphocytes and CD11c^+^ dendritic cells in the skin-draining lymphoid tissues, leading to hyperplasia of the organ at a time when no proliferation of these cells is observed, indicating a local sequestration of leukocytes from peripheral lymph nodes (Demeure et al., 2005).

These findings demonstrate that the subcutaneous transmission of the parasite by the vector potentially generates all of the requirements for priming the host adaptive immune responses in skin-associated tissues. However, no study has demonstrated how the antigens are processed in the tissue-nature source of the antigen-presenting cells that are retained in the skin-draining lymphoid organs after vector transmission of *Plasmodium* sporozoites. It is possible that the accumulated dendritic cell population in the skin-associated draining lymph nodes, which is critical for the priming of protective CD8^+^ T cells against *Plasmodium* sporozoites, is derived from the cutaneous sites where the parasites are inoculated during a mosquito bite. All dendritic cell subsets of skin-draining lymph nodes are defined by the expression of the CD205, CD11b, CD11c, and CD8 markers, including a presumably blood-borne lymph node resident, CD8^+^CD207^+^ Langerhans cell population (Henri et al., 2010; Fehres et al., 2013). It remains unclear whether this cell population can transport antigens to lymph nodes and promote the initiation of T cell responses or, alternatively, if the parasite or its antigens can directly reach the skin-draining lymph nodes that are drained through the conduit network.

Recent studies using CD8^+^ T cell transgenic mice specific for a MHC class I epitope present in the immunodominant surface protein of the sporozoite, the CSP, indicate that, in fact, the first signal of priming for the parasite-specific CD8^+^ T cell response originates from the skin-draining lymphoid tissues associated with the site of vector transmission where the *Plasmodium* sporozoites are also found (Yamauchi et al., 2007). Within the first 48 h, these epitope-specific CD8^+^ T cells secrete IFN-γ in lymph nodes draining the site where the parasite was injected either by a vector bite or by dermal needle injection of the *Plasmodium* parasites (Chakravarty et al., 2007; Sinnis and Zavala, 2012; Ménard et al., 2013). However, in these experimental model studies, the parasites used in the infection were attenuated by irradiation, a matter that could change its infectivity in the skin because it has been shown that potent gliding motility is crucial for sporozoite exit from the cutaneous tissue (Vanderberg, 2014). These studies highlight the importance of the skin in the early stage of infection during the priming of the protective CD8^+^ T cell responses that ultimately will limit the success of the parasite to reach the liver, where its multiplies and establishes its reservoir to continue to the blood-phase of infection.

## The hepatic environment as a specialized niche constituted with professional antigen-processing cells for MHC class I antigens

Studies have revealed that the time from the infective bite to the arrival of the parasite in the liver is approximately one to a few hours (Vanderberg, 2014). During this phase, the parasites must be prepared for the grand finale moment that culminates in the invasion of hepatocyte. Microarray analysis of the sporozoite transcriptome indicated a differential gene expression profile in the salivary gland forms compared to the sporozoites co-cultured with hepatocyte cells. In these studies, 21 genes were confirmed to be up-regulated in sporozoites co-cultured with hepatocytes. These genes were clustered into two patterns of expression, one transiently up-regulated and the other with sustained, increased expression (Rosinski-Chupin et al., 2007; Mikolajczak et al., 2008). These different expression profiles indicate genes that express sporozoite surface proteins that are involved in hepatocyte invasion and transiently expressed and other sustained-expression genes that are involved in the development of parasites inside hepatocytes (Mikolajczak et al., 2008).

Recently, it has been determined that, in fact, there is a shift in the sporozoite forms from the arthropod vector to the mammalian host that is responsible for the activation of the steady-state form of the parasite to its adaptation to the hepatic stages (Coppi et al., 2011). Migrating sporozoites switch to an invasive phenotype in the vertebrate host to reach and colonize the liver. This is well demonstrated in the context of the circumsporozoite protein, which is required for the interaction of the parasite with hepatocyte cells. In salivary glands, the CSP expressed by the sporozoites has a masked domain at its carboxy terminus. During its adaptation in the vertebrate host, the CSP is proteolytically processed in the liver tissue, thus exposing its C-terminal domain, which binds to heparan sulfate proteoglycans on the hepatocyte membrane as the parasite contacts these target cells during hepatic invasion (Coppi et al., 2011). This process is responsible for changing the status of the sporozoite into a hepatic invasive form, which contrasts from the skin form in that the cell transversal activity is the most important mechanism responsible for parasite invasion.

In the liver parenchyma, the parasite needs to glide through tissues and transverse hepatocytes (Ishino et al., 2004). However, this mechanism is not required for final hepatocyte invasion of the parasite before entering the intra-hepatic developmental stage (Ishino et al., 2004, 2005; Kariu et al., 2006; Amino et al., 2008). The invasion mechanism for this step has been shown to be dependent on the recognition of receptors for the region II-plus of the CSP on the basolateral domain of the plasma membrane of hepatocytes (Cerami et al., 1992) and the expression of genes such as the RON4 and the trombospondin-related adhesive proteins TRAP, TREP, TLP, and SPATR, which have roles in sporozoite motility and binding to hepatocyte cells (Sultan et al., 1997; Moreira et al., 2008; Combe et al., 2009). Inside hepatocytes, the sporozoites reside adjacent to the nucleus, and it has been suggested that this topological location may be useful for the parasite's control of the host cells, which occurs by translocation of the CSP into the host nucleus to modulate hepatocyte gene transcription (Singh et al., 2007). During the liver stage, the *Plasmodium* is highly metabolically active and expresses several genes to support its growth and development to blood-stage merozoites (Duffy et al., 2012). It has been shown that the gene products of liver-stage sporozoites are accessible to the host MHC class I-dependent antigen-processing machinery that is required for CD8^+^ T cell recognition and are thus considered potential vaccine targets against the disease (Birkett et al., 2013). Among these genes, the immunogenic properties of *Plasmodium* liver-stage antigen-1 have been investigated, and this antigen has been currently evaluated in vaccine protocols aimed at inducing protection from malaria liver-stage parasites (Hill et al., 1992; Pichyangkul et al., 2008; Rodríguez et al., 2009; Cummings et al., 2010).

The choice of liver-stage development genes as vaccine targets seems to be of relevance because the antigens can be expressed early or late during parasite development in the liver, thus varying the efficacy of the immunity to the infected hepatocytes. However, the paucity of liver-stage, parasite-specific epitopes that are recognized by CD8^+^ T cells has constrained vaccine development approaches. Several preclinical evaluations strongly support the importance of CSP-based vaccine approaches to confer protection mediated by CD8^+^ T cells, but this remains to be determined in humans (Zavala et al., 1985; Khusmith et al., 1991; Kumar et al., 2006). Recently, studies using genome-wide epitope profiling have identified new potential antigen targets in the TRAP (Hafalla et al., 2013). These epitope-mapping studies should improve the design of vaccine protocols based on recombinant viral vectors and DNA vaccines encoding the entire TRAP antigen sequence, which have been shown to induce a protective-mediated immune response against malaria liver-stage infection (Ewer et al., 2013; Ferraro et al., 2013; Hafalla et al., 2013).

Importantly, it has been shown that, besides the early expressed antigens by liver-stage sporozoite forms, such as the TRAP and CS genes, late antigens expressed during the intra-hepatic development of blood-stage merozoites inside hepatocytes are also targets of the host-protective CD8^+^ T cell responses. Virus-vector vaccines expressing the AMA1 from *Plasmodium* parasites induce sterile protection associated with cell-mediated immunity (Chuang et al., 2013; Schussek et al., 2013; Schwenk et al., 2013). AMA1 is a highly conserved antigen in all apicomplexa parasites and is expressed in the merozoite stage of the *Plasmodium* life cycle (Remarque et al., 2008). During the intra-hepatic stage of *Plasmodium* parasites, this antigen is expressed in the late-stage of development of sporozoite forms to the merozoite blood stage. In mice, vaccine-induced AMA1-specific CD8^+^ T cells are associated with sterile protection against experimental malaria infection. In humans vaccinated with a combined genetic vaccine expressing AMA-1 and CSP of *P. falciparum*, 4 of the 15 volunteers (27%) were sterile protected against infection caused by infected mosquito bites (Chuang et al., 2013). Based on these findings, it is worthy to speculate that a better approach for vaccine design should combine the use of antigen targets expressed during the early stages of parasite infection in hepatocytes, e.g., the CSP and TRAP antigens, with the late-expressed AMA1 antigen from *Plasmodium* parasites to potentiate the effectiveness of a vaccine against malaria liver-stage infection.

The exposure of infected hepatocytes to sporozoites developing antigens in the course of infection has been proposed as important for the acquisition of anti-malaria adaptive immune responses, but the basis of hepatocyte antigen processing and presentation are not well understood. Recent studies have investigated the effects of parasite replication on the MHC class I pathway in hepatocytes. A HC-04 cell line isolated from primary human hepatocytes supports the full development of *Plasmodium falciparum* and *Plasmodium berghei* sporozoites to exoerythrocytic merozoite forms in the same time frame as observed *in vivo* (Ma et al., 2013). In these studies, Levitskaya's group has demonstrated that infected human hepatocytes supporting the replication and development of *Plasmodium* parasites to merozoites did not change the mRNA expression of the molecular components of the MHC class I pathway, such as the non-proteolytic (α1 and α2) and proteolytic (β1, β2, and β5) subunits of the proteosome, the elements of the TAP1 and TAP2 that are involved in the non-classical MHC class I cross-presentation pathway of vacuolar antigens, or ER chaperones (calnexin, calreticulin and Erp57) responsible for the complex assembly and stability of MHC class I molecules (Ma et al., 2013). These studies also demonstrated that infected hepatocytes were able to up-regulate the surface expression of MHC class I molecules in response to pro-inflammatory IFN-γ and TNF-α cytokines.

Interestingly, the findings indicated that human hepatocytes infected with *Plasmodium* parasites express the MHC class I processing and presentation machinery of the cross-presenting pathway and are able to stimulate and induce the activation of antigen-specific MHC class I-restricted effector CD8^+^ T cells (Balam et al., 2012). This phenomenon is of relevance because it has been shown that the *in vivo* activation of CD8^+^ T cells specific for CS depends on the cross-presentation of parasite antigens to the class I vacuolar pathway via TAP1 (Cockburn et al., 2011). However, it is important to note that, as the CS protein is an early antigen expressed by the sporozoite liver stages, it is possible that the antigen-processing restriction for the antigens expressed in the late-phase, intra-hepatic developmental stages of *Plasmodium* parasites may have different restrictions and pathways.

**Figure 1:**
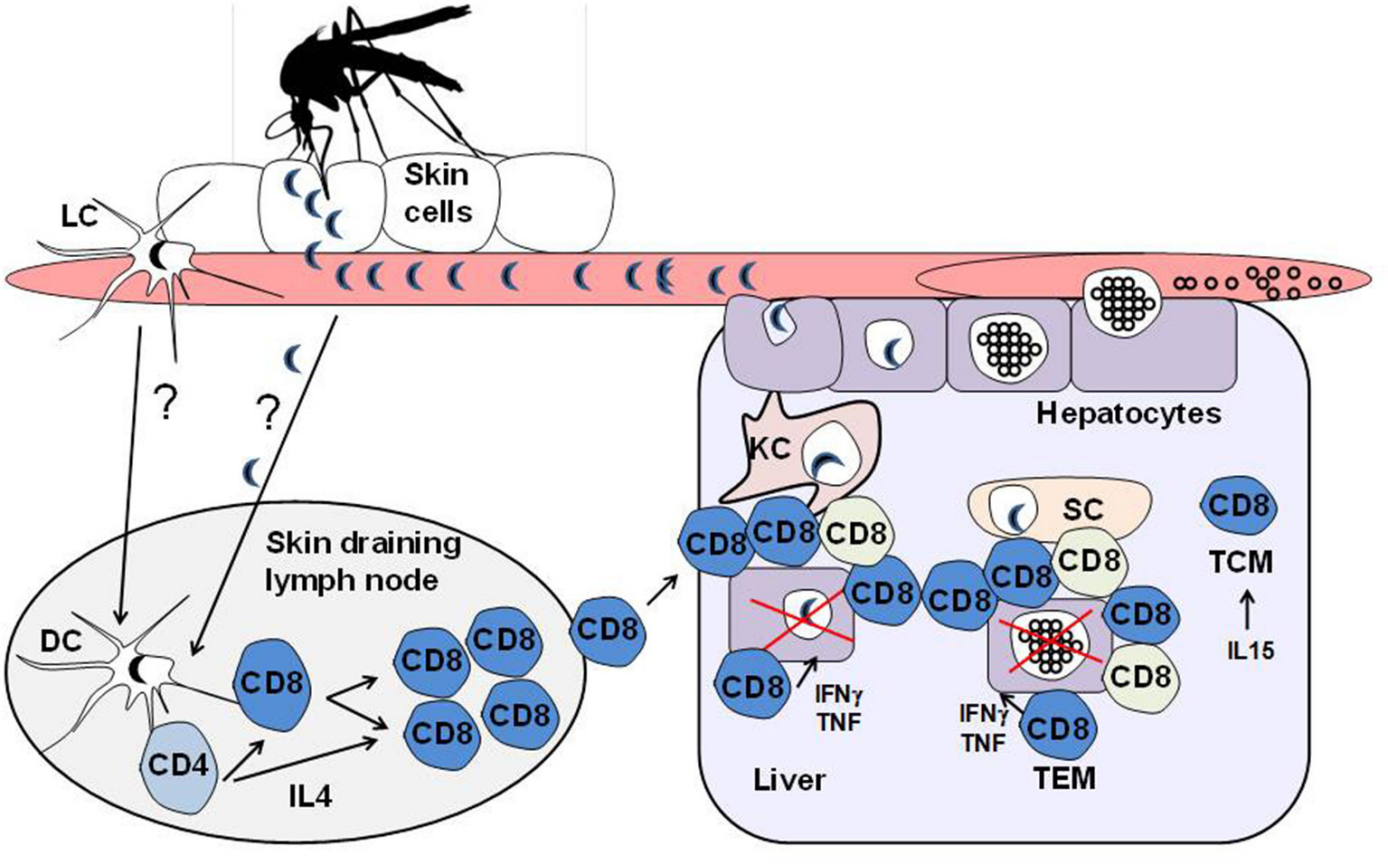
**Model depicting the activation of CD8^+^ T cells against *Plasmodium* liver-stage sporozoites**. Malaria is an infectious disease that begins with the bite of an infected female Anopheline mosquito carrying the sporozoite forms, which is the pre-erythrocytic liver stage of *Plasmodium* parasites. *Plasmodium* sporozoites are released from the secretory duct, where they develop, and are injected into the bite site of the skin during blood meals. The motile sporozoites can then actively disseminate through the skin, bloodstream and draining lymphoid tissues. It is thought that the dendritic cell population in the skin-associated draining lymph nodes, possibly derived from the cutaneous sites where the parasites are inoculated during the vector bite, is critical for the priming of protective CD8^+^ T cells against the *Plasmodium* sporozoites, which are shown to be dependent on IL4-secreting CD4^+^ T helper cells. Although the epidermis and draining lymphoid tissues are thought to initiate the priming of anti-parasite CD8^+^ T cells, these effector cells can undergo further developmental steps in the liver following stimulation with parasite antigens from sporozoites as they develop into the erythrocytic merozoite forms of Plasmodium parasites in the hepatic parenchyma. It has been shown that the hepatic sinusoids are enriched with cells that are able to cross-present MHC class I antigens and therefore act as a barrier that is specialized to process and present antigens to intrahepatic CD8^+^ T cells. The liver antigen-presenting cells include dendritic cells, sinusoidal endothelial cells as well as Kupffer cells. The vast repertoire of antigen-presenting cells found in the journey of the parasite from the skin to the liver could be a determinant for generating distinct CD8^+^ T cell subsets during infection. In fact, it has been shown that effective protection against malaria liver stages is associated with distinct, intra-hepatic immune responses that are characterized by the induction of CD8^+^ T cell subsets with differences in the gene expression profiles associated with the cell trafficking responses that could play roles in the local immunity by tissue resident memory/effector cells. In the figure, the different colored CD8^+^ T cells represent the heterogeneous effector populations found in the liver following sporozoite infection.

The cross-presentation MHC class I pathway in the liver seems to be extended to different subsets of hepatic cells, and this function was observed in liver sinusoidal endothelial cells as well as Kupffer cells at low antigen concentrations (Ebrahimkhani et al., 2011). Interestingly, the antigen cross-presentation by liver cells induced efficient CD8^+^ T cell expansion comparable to classical dendritic cells obtained from the spleen (Plebanski et al., 2005; Ebrahimkhani et al., 2011). However, T cells that proliferated following activation by contact with antigen-presenting liver cells expressed lower levels of T cell activation markers and intracellular IFN-γ, suggesting that dendritic cells may have a role in compensating the full activation program of CD8^+^ T cells that are initially activated in the hepatic parenchyma (Ebrahimkhani et al., 2011), a scenario that could support the full priming of CD8^+^ T cells in the liver. Alternatively, it is possible that the primary CD8^+^ T cells activated in the draining lymphoid organs during skin infection may support further activation and differentiation by liver cells once the parasite targets the hepatic parenchyma during malaria infection (Figure 1).

## CD8^+^ T cells against malaria liver stages are defined as different subsets and mediate protection in the course of a natural or vaccination-induced immune response

As in most infections caused by intracellular pathogens, CD8^+^ T cells are essential for developing acquired immunity against malaria liver-stage infection (Shrikant et al., 2010; Gebhardt and Mackay, 2012; Remakus and Sigal, 2013). Several independent investigations have demonstrated a critical role for CD8^+^ T cells in adaptive immunity against the pre-erytrocytic stage of *Plasmodium* infection using different approaches, such as *in vivo* depletion, reconstitution and adoptive transfer studies (Doolan and Martinez-Alier, 2006). Former studies in experimental rodent models indicated that immunization with irradiated *P. berghei* and *P. yoelli* sporozoites is able to induce protective immune responses mediated by CD8^+^ T cells, and *in vivo* depletion of this T cell subset abolished the acquired protective immunity (Schofield et al., 1987; Weiss et al., 1988; Seguin et al., 1994; Doolan and Hoffman, 1999, 2000). Although CD8^+^ T cells are considered to be the primary effector cells in the protective immune responses induced by irradiated sporozoites, CD4^+^ T cells specific to parasite-epitope antigens associated with MHC class II complexes expressed by infected hepatocytes are also thought to exert a role in host protection (Weiss et al., 1993).

Depending on the mouse strain used in the experimental rodent model of malaria infection, there is a requirement for CD4^+^ T cells in the effector responses induced by immunization with irradiation-attenuated sporozoites. It has been shown that C57BL/6, B6.129, and B10 mice immunized with irradiated sporozoites and previously depleted of CD4^+^ T cells were not protected against sporozoite challenge (Doolan and Hoffman, 2000; Doolan and Martinez-Alier, 2006). These results indicate a critical role for CD4^+^ T cells in the protective, immune-mediated responses against malaria liver-stage sporozoites. However, this CD4^+^ T cell requirement was not observed when BALB/c, B10.BR, and A/J mice were infected (Doolan and Hoffman, 2000). The CD4^+^ T cell requirement indicates a helper function-dependence for the induction or maintenance of effector CD8^+^ T cell responses, similar to how it occurs in other models. This dependence was further investigated using CD8^+^ T cell receptor (TCR) transgenic mice against the SYIPSAEKI epitope from the *Plasmodium yoelli* CSP antigen (Carvalho et al., 2002).

In the TCR transgenic system, transferring of CSP-specific, transgenic CD8^+^ T cells into normal mice subsequently immunized with irradiation-attenuated sporozoites can induce a protective immune response against sporozoite challenge (Sano et al., 2001). This protective response was not observed when CD8^+^ TCR transgenic cells were transferred into normal mice that had been previously depleted of CD4^+^ T cells or IL-4 gene knockout recipient mice (Carvalho et al., 2002). In the absence of CD4^+^ T cells, effector antigen-specific CD8^+^ T cells begin a normal activation program with proliferative expansion in the first stages of the T cell differentiation program. However, after this initial phase, CD8^+^ TCR transgenic cells activated in the absence of CD4^+^ T helper cells fail to develop further, which compromises the effector pool of antigen-specific CD8^+^ T cells, as represented by a reduced number of cells and lower frequencies of IFN-γ secreting, antigen-specific TCR T cells. Using adoptive transfer and reconstitution approaches with gene-knockout cells to dissect the CD4^+^ T cell helper function, studies on the CSP-specific CD8^+^ TCR transgenic system indicated a requirement for the interleukin loop involving IL-4 receptor expression on CD8^+^ cells and that IL-4 secreted by CD4^+^ helper T cells was necessary for the full development of antigen-specific CD8^+^ T cells in the protective cellular-adaptive response against *Plasmodium* liver-stage parasites (Carvalho et al., 2002; Morrot et al., 2005).

Many studies of malaria as well as listeriosis and hepatitis models have raised attention to the complexity of the adaptive immune response in the hepatic microenvironment (Shrikant et al., 2010; Condotta et al., 2012; Gebhardt and Mackay, 2012; Claassen et al., 2013; Remakus and Sigal, 2013). Although these studies point to a central role for CD8^+^ effector/memory T cells in the elimination of infectious pathogens in the liver, the cellular and molecular interactions that underscore the mechanisms leading to pathogen killing are still poorly understood. In malaria, *in vivo* studies have investigated the dynamics of effector CD8^+^ T cells in the hepatic microenvironment. The immunization of naïve BALB/c mice with radiation-attenuated sporozoites significantly increased the presence of CD8^+^ T cells patrolling the sinusoids (Guebre-Xabier et al., 1999; Cabrera et al., 2013). Importantly, studies using the TCR CSP-specific transgenic system have approached intravital dynamic imaging of *Plasmodium* elimination and revealed that parasite elimination frequently involves the recruitment of antigen-specific CD8^+^ T cells to the liver parenchyma and the spatial distribution of these cells in a cluster surrounding the infected hepatocytes. These clusters of parasite-specific CD8^+^ T cells reveal an efficacious, cell-mediated protective immunity mechanism against antigen targets expressed by sporozoite forms during malaria liver-stage infection (Cockburn et al., 2013).

Multiple effector mechanisms are likely to mediate *Plasmodium* liver-stage elimination by CD8^+^ T cells (Frevert and Nardin, 2008). The protective effector mechanisms exerted by CD8^+^ T cells in the pre-erythrocytic malaria stages in infected or vaccinated hosts varies depending on the infected animal model or vaccination regimen (Doolan and Martinez-Alier, 2006; Frevert and Nardin, 2008; Duffy et al., 2012). Although the literature has advanced our knowledge of protective host CD8^+^ T cell immunity in rodent models, little is known about the cellular events that occur in the intrahepatic malaria stage of naturally infected humans. CD8^+^ T cells can induce infected host cell killing by redundant mechanisms in which IFN-γ and TNF-α are identified as important players of the non-cytolytic pathways of infected hepatocytes during liver-stage infection of *Plasmodium* parasites (Butler et al., 2010). Another classical mechanism is mediated by the granule exocytosis pathway, which is a common characteristic of CD8^+^ T cells and NK cells. Cytotoxic granules are a product of the secretory lysosomes that contain the pore-forming proteins perforin and granulysin, which are granzymes that mediate apoptosis in target cells (Frevert and Nardin, 2008). A preclinical trial of an optimized DNA vaccine approach that targets multiple sporozoite and liver-stage antigens, including CSP, LSA1, TRAP, and CelTOS, demonstrate the acquisition of antigen-specific CD8^+^ granzyme B^+^ T cells in non-human primates (Ferraro et al., 2013). The elimination of *Plasmodium* liver-stage parasites is likely to be mediated by direct recognition of infected hepatocytes by antigen-specific CD8^+^ T cells, as this event occurs without a bystander effect that could promote parasite killing over the distance from the synapse that is induced between these cells in the liver parenchyma (Cockburn et al., 2014).

The multiple effector mechanisms deployed by CD8^+^ T cells against the pre-erythrocytic-stage of the *Plasmodium* parasite is suggestive of a possible different commitment of the intrahepatic CD8^+^ T cell lineage in malaria liver-stage infection. These cells exhibit a transcriptional profile with a distinguishable expression of immune function genes, cell cycle control and cell trafficking (Tse et al., 2013). In fact, it has been shown that effective protection against malaria liver stage is associated with distinct, intra-hepatic immune responses that are characterized by the induction of different CD8^+^ T cell subsets. Studies have demonstrated that the protective T cells that are induced by attenuated malaria parasites induce changes in the CD8^+^ T cell population, which is characterized by upregulation of CD11c on effector CD3^+^CD8^+^ T cells in the liver, spleen and peripheral blood. The majority of these cells are CD11c^hi^CD44^hi^CD62L^−^ and secrete pro-inflammatory cytokines and cytotoxic markers such as IFN-γ, TNF-α, interleukin-2, perforin and CD107a. CD11c expression is lost as the CD8^+^ T cells progress to memory phase (Cooney et al., 2013).

Other studies have demonstrated that vaccine-induced immunity using attenuated *Plasmodium* sporozoites is accompanied by the presence of intrahepatic effector memory (EM) CD8^+^ T cells characterized by a CD44^hi^CD45RB^lo^CD62L^lo^CD122^lo^ phenotype and central memory (CM) CD8^+^ T cells with a distinguished phenotype, CD44^hi^CD45RB^hi^CD62L^hi^CD122^hi^, that are maintained by IL-15-mediated homeostatic proliferation (Krzych and Schwenk, 2005). The EM CD8^+^ T cells promptly secrete IFN-γ upon sporozoite challenge, and these intra-hepatic memory CD8^+^ T cells can be boosted by re-exposition to sporozoite antigens (Krzych and Schwenk, 2005). This feature could be of relevance, considering that it has recently been shown that the liver environment can keep a persisting depot of liver-stage antigens from irradiated sporozoites over 8 weeks after immunization, which is required for optimal development of protective immune responses mediated by CD8^+^ T cells (Cockburn et al., 2010).

The presence of different T cell memory subsets may implicate a self-competition of CD8^+^ T cells for the antigen, which, in turn, would limit the expansion/magnitude of the EM and CM pools of memory CD8^+^ T cells (Hafalla et al., 2002, 2003; Cockburn et al., 2010). This intricate, self-regulatory mechanism exerted by activated CD8^+^ T cells may have implications for the development of malaria liver-stage vaccines, considering it has been demonstrated that a large threshold for memory CD8^+^ T cell frequencies is required for long-term protection. In these studies, the authors have developed a model of epitope-specific immunization regimes to induce a large memory CD8^+^ T cell response capable of protecting mice from sporozoite challenges (Schmidt et al., 2008). Although the issue concerning the persistence of parasite antigens for the maintenance of memory T cells still remains elusive as some studies have demonstrated that primaquine treatment of immunized mice with irradiated sporozoites does not affect the protective responses (Krzych et al., 2014). However, since the primary primaquine action is target to the pre-erytrocytic liver stage parasite development, its effect does not interfere with the tissue depot of sporozoite antigens responsable for the activation/maintenance of host specific T cells (Krzych et al., 2014). Although the supporting mechanisms responsible for the persistence of memory T cells remains still controversial, a robust and sustainable intrahepatic CD8^+^ T cell response is the target of the vaccine designs aimed at avoiding the intrahepatic development of malaria blood-stage merozoite forms, which are ultimately responsible for the clinical signs of the disease. It is possible that varying the route and/or nature of the protective antigen regimen in the prime-boost strategy implied in the vaccine protocols would overcome this restriction. This is of particular relevance and should be taken into consideration because sustainable protection against malaria is characterized by acquisition of strong, IFN-γ-secreting, intrahepatic CD8^+^ memory T cells (Nganou-Makamdop et al., 2012).

### Development of a recombinant vaccine against the pre-erythrocytic stage of malaria

The only malaria vaccine that has reached Phase III clinical trials consists of a recombinant version of the *P. falciparum* CSP that is administered in an adjuvant system. The efficacy of this vaccine formulation is approximately 30–50% and greatly correlates with the antibody titers to *P. falciparum* sporozoites and, to a minor extent, to CSP-specific CD4^+^ T cells capable of secreting two or more cytokines (Birkett et al., 2013). Because this vaccine formulation already elicits antibodies and CD4^+^ T cell-mediated immunity, to improve the vaccination efficacy, simultaneous stimulation of effector mechanisms mediated by CD8^+^ T cells can be useful. Currently, the heterologous prime-boost regimen has achieved the best results in terms of a strong, protective immune response mediated by CD8^+^ T cells specific for the pre-erythrocytic stages of malaria parasites (Hill, 2011; Soares et al., 2012; Teixeira et al., 2014).

Unfortunately, the results obtained after heterologous prime boost vaccination in a mouse model have not been duplicated in non-human primates or in humans (Birkett et al., 2013). Most of the studies rely on the analysis of the protection efficacy from the different vaccine regimens (Hill et al., 2010; Hill, 2011; Birkett et al., 2013). As the protection against malaria liver stage directly correlates with the acquisition of strong IFN-γ secreting intrahepatic CD8^+^ T cells (Nganou-Makamdop et al., 2012), the levels of these responses elicited in the different vaccines would be an important parameter to guide the efficiency of a vaccine design. However, due to the absence of a direct method to identify and phenotype malaria vaccine-induced intrahepatic CD8^+^ T cells in humans and non-human primates during pre-clinical and clinical tests, it should be important to determine whether the levels of antigen-specific CD8^+^ T cells found in the liver correlates with the peripheral CD8^+^ T cell responses from PBMC blood found in murine models, using a subunit vaccine for proof-of-concept.

The results of clinical trials using the heterologous prime-boost vaccination regimen with the *pfcsp* and *pfama-1* genes were recently published (Hill et al., 2010). This protocol consisted of priming with recombinant plasmid DNA, followed by a booster immunization with AdHu5, both expressing the *pfcsp* and *pfama-1* genes from the 3D7 strain of *P. falciparum*. Upon experimental challenge by exposure to the bites of mosquitos infected with the homologous parasite strain, only 27% of the individuals were sterilely protected (Bruder et al., 2012).

In other studies, individuals were initially immunized with recombinant adenoviral vector type 63 from chimpanzees that contained a synthetic gene encoding the polypeptide denominated ME-TRAP (Sheehy et al., 2012). The individuals received boost vaccinations with a recombinant MVA also containing this same gene (Bejon et al., 2007). In additional heterologous prime boost Phase II studies, 3 of 14 individuals were sterilely protected from infection after exposure to *P. falciparum*-infected mosquitoes (Bejon et al., 2007).

### Development of an attenuated parasite vaccine against the pre-erythrocytic stage of malaria

Although it has been long established that attenuated sporozoites provide a high degree and relatively long-lived protective immunity in most hosts, including men, vaccination regimens using whole, attenuated sporozoites were, until recently, considered a difficult path for the development of a product for mass vaccination. The recent development of cGMP *P. falciparum* sporozoite cultivation methods has now made this path a tangible possibility (Epstein et al., 2011). This strategy offers several advantages. Attenuated sporozoites express and present the whole spectrum of immunogens associated with the sporozoite- and liver-stages of malaria to the host (Butler et al., 2011; Epstein et al., 2011; Duffy et al., 2012; Spring et al., 2013). As the attenuated parasite is still able to infect target tissues, such as skin and liver parenchyma, in its natural route of infection, it induces the development of specific antibodies, CD4^+^, CD8^+^, and γδ T cells. This wide range of immunogens and mechanisms of defense has a better chance of eliminating all pre-erythrocytic stages, which is required for sterile protection. In agreement with this idea, a recent study showed that protocols using multiple intravenous injections of radiation-attenuated sporozoites could provide sterile, protective immunity to 100% of vaccinated individuals (Seder et al., 2013). This observation may lead to a rational strategy for the development of a vaccine against malaria infection. Although important challenges should be overcome to face a large scale generation of *Plasmodium* sporozoites. To this goal, the development of aseptic, purified and cryopreserved *Plasmodium falciparum* sporozoites has received important priority (Seder et al., 2013). Improvements to stabilization of cryopreserved sporozoites are critically important to maintain high infectivity for the development of a vaccine using radiation-attenuated sporozoites. This is particularly critical to reduce vaccine wastage by prolonging the time after thawing the cryopreserved *Plasmodium* parasites during the vaccination.

## Summary and perspectives

The skin tissue acts as natural barrier against invading pathogens. The recent advances in skin immunobiology and studies pointing to the importance of the skin for the induction of the first signs of an anti-*Plasmodium*-adaptive responses induced by CD8^+^ (Sinnis and Zavala, 2012)T cells against whole sporozoite forms in natural infections have substantiated a new concept in malaria biology and will consolidate the efforts for designing an efficacious vaccine against the pre-erythrocytic stages (Ménard et al., 2013). Recent advances in rodent models indicate a parasite skin-developmental pathway leading to generation of merozoites in the skin hair follicles that are infective to erythrocytes (Gueirard et al., 2010). This feature is able to promote a shortcut in the malaria cell cycle and should reshape all the current goals for malaria vaccine development strategies which target the induction of immunity to prevent the clinical signs of disease and/or interrupt transmission to support the eradication of this infirmity. Vaccines aimed at targeting the pre-erythrocytic stages of *Plasmodium* parasite infection can reach the spectrum of the vaccine goals against malaria, as any intervention at the pre-erythrocytic stage of the parasite cycle would have an implication in the further development of the parasite to the blood-associated disease symptoms and the sexual-stage parasites that are required for vector transmission.

### Conflict of interest statement

Maurício M. Rodrigues is named inventor on patent applications entitled “PLASMODIUM VIVAX VACCINE COMPOSITIONS” File reference no.:27522-0201WO1. The authors declare that the research was conducted in the absence of any commercial or financial relationships that could be construed as a potential conflict of interest.

## Abbreviations

PAMPs: Pathogen-associated molecular patterns
GPI: Glycosylphosphatidylinositol
TLR: Toll-like receptor
CSP: Circumsporozoite protein
RON4: Rhoptry resident protein
TRAP: Thrombospondin-related adhesion protein
ME-TRAP: Multiple epitope-thrombospondin-related adhesion protein
TLP: TRAP-Like Protein
TREP: TRAP-related protein
SPATR: Surface protein containing an altered thrombospondin repeat domain
AMA1: Apical membrane antigen 1
TAP: Vacuolar complex peptide transporters
LSA1: Liver-stage antigen 1
CelTOS: Cell-traversal protein for sporozoites
MVA: Modified vaccinia Ankara virus
AdHu5: Human type 5 replication-deficient adenovirus
TEM: Effector memory T cells
TCM: Central memory T cells
DC: Dendritic cell
LC: Langerhans cell
SC: Sinusoidal endothelial cells
KC: Kupffer cell.
